# Does metabolic control of the disease related with bone turnover markers in children with type 1 diabetes mellitus in Turkey?

**DOI:** 10.1186/s12902-024-01553-0

**Published:** 2024-06-14

**Authors:** Merve Sena Topkaya, Onur Akın, Tuğba Küçükkasap Cömert

**Affiliations:** 1 Department of Nutrition and Dietetics, Gülhane Health Sciences Institute, Health Sciences University, Ankara, Turkey; 2 Specialist of Pediatric Endocrinology, Department of Pediatric Endocrinology, Gülhane Training and Research Hospital, Ankara, Turkey

**Keywords:** Type 1 diabetes mellitus, Oxidative stress, Bone biomarkers, Metabolic control

## Abstract

**Background:**

The aim was to evaluate the effect of metabolic control on bone biomarkers in children with type I diabetes.

**Materials and methods:**

The children were divided into two groups according to their glycated hemoglobin (HbA1c) (%) levels: a group with HbA1c levels < 8% (*n* = 16) and: a group with HbA1c levels > 8% (*n* = 18). The serum total oxidative status (TOS) (µmol/L), total antioxidant status (TAS) (mmol/L), alkaline phosphatase (ALP) (IU/L), osteocalcin (OC) (ng/ml), procollagen type-1-N-terminal peptide (P1NP) (ng/ml), and vitamin D (IU) levels and food consumption frequencies were determined.

**Results:**

When patients were classified according to HbA1c (%) levels, those with HbA1c levels < 8% were found to have lower TOS (µmol/L) values (8.7 ± 6.16, 9.5 ± 5.60) and higher serum OC (ng/mL) (24.2 ± 16.92, 22.0 ± 6.21) levels than those with HbA1c levels > 8% (*p* < 0.05). Regardless of the level of metabolic control, there was a statistically significant association between serum TOS (µmol/L) and P1NP (ng/ml) (*p* < 0.05) levels, with no group-specific relationship (HbA1c levels <%8 or HbA1c levels >%8).

**Conclusion:**

HbA1c and serum TOS levels had an effect on bone turnover biomarkers in individuals with type I diabetes.

## Background

Type 1 diabetes mellitus (T1DM) is a chronic disease that requires insulin treatment due to the destruction of pancreatic beta-cells and can affect bone and skeletal health [1, 2]. Low bone mineral density, bone fractures, and delayed healing of fractures are reported problems in children and adults with T1DM [3]. The bone cycle is a balanced and coordinated relationship between osteoblasts responsible for bone turnover and osteoclasts responsible for resorption [4]. Alkaline phosphatase (ALP) and osteocalcin (OC) are generally used as bone formation turnover markers [3]. In recent years, the International Osteoporosis Foundation has proposed procollagen type-1 N-terminal propeptide (P1NP), which originates from osteoblasts and occurs during collagen type 1 formation, as a more specific marker of bone turnover [5].

Bone changes associated with T1DM can be explained by various mechanisms, including altered calcium and vitamin D metabolism due to hyperglycemia, glycation of type I collagen in bone, low insulin-like growth factor-1 (IGF-1) levels, accumulation of lipids in bone marrow, and increased oxidative stress [6].

OS is characterized by an imbalance between the generation of free radicals and the body’s antioxidant status. OS is increased in T1DM patients as a result of hyperglycemia through different pathways, increased production of reactive oxygen species (ROS) in mitochondria, glucose auto oxidation, enhanced polyol pathways, and the formation of glycation end products (AGEs) [2]. Oxidative stress can negatively affect bone by increasing bone resorption by osteoclasts and inhibiting osteoblastic differentiation. Furthermore, ROS increase lipid peroxidation by increasing malondialdehyde (MDA), the end product of lipid peroxidation [7]. Therefore, it is hypothesized that diabetes-induced overproduction of reactive oxygen and nitrogen species may play a critical role in the biological performance of bones in diabetic patients [8].

In the literature, studies [9–11] have evaluated bone turnover biomarkers in healthy children and children with T1DM. In this study, individuals with T1DM were evaluated in two groups (HbA1c levels < 8% and HbA1c levels > 8%) according to their target HbA1c levels in line with the American Diabetes Association recommendations [12], and we aimed to evaluate the effects of metabolic control, diet compliance, food consumption, and serum total oxidative status (TOS) on bone turnover markers. We hypothesized that high HbA1c and serum TOS levels would be associated with lower bone turnover markers and diet would be a contributing factor to this situation in children with T1DM.

## Methods

### Individuals

The study was conducted on a total of 34 children aged 3–17 years who were diagnosed with T1DM at least one year ago. The study sample consisted of a group that was followed up at a pediatric endocrinology and metabolism clinic and whose diagnosis and treatment were carried out by an endocrinologist. According to the American Diabetes Association [12] recommendations (fasting blood glucose level > 126 mg/dL or postprandial blood glucose level > 200 mg/dL), type 1 diabetes was diagnosed by an endocrinologist. Children who (a) were taking medication that could affect glucose metabolism or bone parameters, (b) were diagnosed with malabsorption, (c) had a history of bone fracture, (d) had a secondary chronic disease, (e) had acute illness at the time of data collection, and (f) had 85th percentile ≤ BMI values < 95th percentile (overweight) and > 95th percentile (obese) [13] were not included in the study.

When the sample calculation was performed by using power analysis with a power of 0.95 and a significance level of 0.05, with reference to Table 1 in the study by Amrousy et al. [10], it was determined that at least 15 diabetic children per group and at least 30 diabetic children in total were needed for this study.

**Table 1:** Age, anthropometric measurements, HbA1c levels and years with DM according to HbA1c (%) level

Variable	HbA1c <%8 (*n* = 16)						HbA1c >%8 (*n* = 18)						TOTAL (*n* = 34)						*p*
	x ± SD	Median	Q1	Q2	Q3	Q4	x ± SD	Median	Q1	Q2	Q3	Q4	x ± SD	Median	Q1	Q2	Q3	Q4	
**Age**	9.9 ± 3.77	9.0	7.0	9.0	12.7	15.0	10.5 ± 2.30	11.0	8.7	11.0	12.0	16.0	10.0 ± 3.08	10.5	7.7	10.5	12.0	16.0	0.313
**Weight (kg)**	35.7 ± 14.88	32.9	25.2	32.9	47.9	64.0	39.6 ± 9.98	41.3	32.4	41.3	46.6	58.0	37.8 ± 12.48	35.1	26.9	35.1	46.6	64.0	0.297
**Height (cm)**	139.0 ± 22.80	133.3	126.5	133.3	158.7	182.0	144.9 ± 14.61	147.0	135.7	147.0	158.7	170.0	142.1 ± 18.85	140.1	130.2	140.1	158.7	182.0	0.330
**BMI (kg/m** ^**2**^ **)**	17.6 ± 2.05	17.9	15.4	17.9	19.3	20.7	18.6 ± 1.70	18.8	17.0	18.8	19.6	22.5	18.1 ± 1.90	18.4	16.6	18.4	19.4	22.5	0.164
**HbA1c (%)**	7.11 ± 0.53	7.1	6.7	7.1	7.5	7.9	9.4 ± 1.27	8.8	8.4	8.8	10.1	12.2	8.3± 1.51	8.1	7.1	8.1	9.0	12.2	0.000*
**Years with DM**	5.0 ± 3.36	4.5	2.5	4.5	6.5	13.0	5.5 ± 2.70	6.0	2.7	6.0	8.0	10.0	5.2± 2.99	5.0	2.7	5.0	8.0	13.0	0.384

*p < 0.05

Children who met the inclusion criteria were divided into two groups according to their HbA1c level in accordance with the American Diabetes Association recommendations [12]: a group with HbA1c levels < 8% (*n* = 16) and a group with HbA1c levels > 8% (*n* = 18).

### Data collection

The demographic characteristics, dietary habits, food consumption records, and food consumption frequencies of the children were recorded by the researchers through a questionnaire form completed through a face-to-face interview, and HbA1c values were obtained from the hospital electronic database.

### Biochemical analyses

Venous blood samples were collected by a nurse in the morning after the participants had fasted for 8 h, and the samples were stored at − 80 °C until analysis. TOS and TAS levels were analyzed using a commercial enzyme-linked immunosorbent assay kit (Relay, Turkey) [14, 15]. Serum ALP, OC, and P1NP levels were evaluated by the enzyme-linked immunosorbent assay (ELISA) method [16–19]. Data on HbA1c and vitamin D levels were obtained from the hospital electronic database and from the HPLC method used in the hospital.

### Dietary analyses

To determine the daily nutrient intake (energy, macronutrient, and micronutrient intake), a semiquantitative food frequency questionnaire (FFQ) validated by Dikmen et al. (2016) [20] was used and analyzed by Bebis version 7.2 software (Ebispro, Stuttgart, Germany) [21].

### Statistical analyses

Statistical analysis was performed using the Statistical Package for Social Sciences (SPSS) version 22.0 for Mac (SPSS Inc., Chicago, IL). Chi-square test was used for comparing qualitative data of the two groups. A comparison of quantitative data of the two groups was carried out using Mann-Whitney U test. The relationship between bone turnover markers and oxidative stress parameters was evaluated using multiple regression analysis. The significance level was determined as *p* < 0.05.

## Results

The table shows the distributions of age, weight, height, BMI, and HbA1c (%) levels in children with type 1 diabetes according to the HbA1c (%) level. The mean age of the children with diabetes who participated in the study was 10.0 ± 3.08 years. The study was conducted on a total of 34 children with T1DM; 58.8% of the children were male and 41.2% were female. The mean age was 10.0 ± 3.08 years, the mean BMI was 18.1 ± 1.90 kg/m^2^, the mean HbA1c level was 8.3 ± 1.51, and the mean number of years with DM was 5.2 ± 2.99. No difference were not found between the groups (Table 2).

**Table 2:** Dietary habits of children according to HbA1c (%) level

Dietary habits		HbA1c<%8 (*n* = 16)		HbA1c>%8 (*n* = 18)		TOTAL (*n* = 34)		
		*N*	%	*N*	%	*N*	%	*p*
**Meal skipping status**	**Yes**	3	18.7	6	33.3	14	41.2	0.339
	**No**	13	81.3	12	66.7	20	58.8	
**Reason for skipping meals**	**Don’t want**	2	66.7	4	66.7	6	66.7	1.000
	**Timelessness**	1	33.3	2	33.3	3	33.3	
**Food preference in snacks**	**Snack**	4	25.0	2	11.1	6	17.7	0.131
	**Milk/fruit**	12	75.0	16	88.9	28	82.3	
**Where they consume their lunch**	**Canteen**	3	18.8	6	33.3	9	29.5	0.408
	**Home/cafeteria**	13	81.2	12	66.7	25	70.5	

It was found that 41.2% of the children skipped meals, 58.8% stated that they did not want to skip meals, and 33.3% stated that they did not consume meals due to time constraints. For snacks, 82.3% of the children preferred milk/fruit, 17.7% preferred snacks, and 70.5% consumed lunch at home/in a cafeteria. When dietary habits were evaluated according to the HbA1c level, there was no significant difference between the groups (Table 1).

When nutrient consumption was evaluated, the mean daily energy consumption was 1686.7 ± 367.99 kcal/day, and protein, fat, and carbohydrate (CHO) consumption was 59.8 ± 14.52 g/day, 74.8 ± 19.34 g/day, and 189.1 ± 61.43 g/day, respectively. When nutrient consumption was evaluated according to HbA1c level, it was found that protein and sodium (Na) consumption in children with HbA1c level < 8% (54.5 ± 14.44 g/day and 2865.1 ± 567.35 mg/day, respectively) was greater than that in children with an HbA1c level > 8% (64.6 ± 13.22 g/day and 2865.1 ± 567.35 mg/day, respectively) (*p* < 0.05), while no significant difference was found between the groups in terms of other nutrients (Table 3).

**Table 3:** Average daily energy and nutrient intakes of children according to HbA1c (%) level

	HbA1c <%8 (*n* = 16)						HbA1c >%8 (*n* = 18)						TOTAL (*n* = 34)						*p*
	x ± SD	Median	Q1	Q2	Q3	Q4	x ± SD	Median	Q1	Q2	Q3	Q4	x ± SD	Median	Q1	Q2	Q3	Q4	
**Energy (kcal)**	1608.6 ± 428.35	1492.7	1232.0	1492.7	1995.0	2359.8	1756.2 ± 300.22	1860.1	1628.3	1860.1	1941.1	2290.9	1686.7 ± 367.99	1751.9	1326.5	1751.9	1941.1	2359.8	0.211
**Protein (g)**	54.5 ± 14.44	53.5	45.5	53.5	55.8	89.9	64.6 ± 13.22	64.6	54.3	64.6	71.3	88.4	59.8 ± 14.52	55.5	52.1	55.5	68.0	89.9	0.017*
**Protein (%)**	14.1 ± 2.61	14.5	12.0	14.5	16.7	18.0	15.3 ± 3.00	14.5	13.7	14.5	17.5	22.0	14.7 ± 2.84	14.5	13.0	14.5	17.0	22.0	0.443
**Fat (g)**	69.1 ± 22.17	68.7	49.7	68.7	88.4	102.4	79.9 ± 15.20	82.8	70.3	82.8	89.5	110.7	74.8 ± 19.34	75.9	60.6	75.9	89.3	110.7	0.126
**Fat (%)**	38.3 ± 7.40	39.5	35.2	39.5	43.7	49.0	41.0 ± 7.18	40.0	35.75	40.0	46.0	57.0	39.7 ± 7.30	40.0	35.7	40.0	45.0	57.0	0.528
**Carbohydrate(g)**	187.8 ± 68.21	181.6	136.3	181.6	210.1	363.2	190.1 ± 56.72	200.9	151.2	200.9	224.8	298.7	189.1 ± 61.43	183.0	146.4	183.0	221.8	363.2	0.597
**Carbohydrate(%)**	47.3 ± 8.67	45.0	41.2	45.0	52.5	65.0	43.7 ± 8.39	43.5	39.5	43.5	52.0	58.0	45.4 ± 8.58	44.0	40.0	44.0	52.0	65.0	0.384
**Dietary fibre (g)**	19.9 ± 6.49	19.8	15.5	19.8	23.9	36.0	18.7 ± 5.87	18.6	15.1	18.6	22.0	32.1	19.3 ± 6.10	19.1	15.5	19.1	22.7	36.0	0.621
**Vitamin A (µg)**	700.6 ± 256.35	745.4	535.5	745.4	928.3	972.5	1061.9 ± 779.86	832.2	663.7	832.2	1300.5	3651.0	891.9 ± 613.76	802.2	618.6	802.2	956.7	3651.0	0.281
**Vitamin E(mg)**	14.5 ± 7.45	16.9	7.3	16.9	18.5	28.8	16.5 ± 8.17	15.0	10.0	15.0	25.4	34.1	15.5 ± 7.79	16.4	8.4	16.4	20.5	34.1	0.484
**Thiamine (mg)**	0.8± 0.25	0.8	0.7	0.8	0.9	1.4	0.9± 0.19	0.9	0.7	0.9	1.0	1.3	0.9 ± 0.22	0.8	0.7	0.8	1.0	1.4	0.297
**Riboflavin(mg)**	1.2± 0.42	1.1	0.9	1.1	1.3	2.4	1.5± 0.32	1.5	1.2	1.5	1.7	2.2	1.3 ± 0.39	1.3	1.0	1.3	1.6	2.4	0.018*
**Niacin (mg)**	10.4 ± 4.39	9.9	6.7	9.9	13.0	20.2	11.5 ± 3.96	10.5	8.4	10.5	15.1	18.5	11.1 ± 4.15	10.5	7.3	10.5	13.9	20.2	0.384
**Vitamin B6 (mg)**	1.2± 0.39	1.3	0.9	1.3	1.5	2.1	1.3± 0.47	1.2	1.0	1.2	1.7	2.5	1.3 ± 0.43	1.2	1.0	1.2	1.5	2.5	0.551
**Folate (µg)**	104.0 ± 44.00	93.9	75.4	93.9	119.0	226.6	123.6 ± 45.98	120.3	101.1	120.3	159.9	214.8	282.6 ± 80.42	115.2	233.0	287.6	341.4	435.1	0.095
**Vitamin B12 (µg)**	4.3 ± 2.51	3.7	2.6	3.7	5.9	10.9	5.3± 2.07	4.7	3.7	4.7	6.3	10.8	4.8± 2.31	4.4	3.4	4.4	6.1	10.9	0.135
**Vitamin C (mg)**	100.6 ± 68.75	80.5	48.4	80.5	154.4	229.8	127.3 ± 95.44	89.4	48.8	89.4	182.9	368.0	114.7 ± 83.81	89.4	50.4	89.4	170.7	368.0	0.528
**Sodium (mg)**	3165.1 ± 567.35	2697.7	2469.3	2697.7	3257.3	4218.1	3622.0 ± 1130.46	3426.8	2740.3	3426.8	3991.5	6289.5	3265.8 ± 975.53	3042.9	2575.4	3042.9	3548.3	6289.5	0.027*
**Potassium (mg)**	2237.7 ± 577.21	2197.5	1998.9	2197.5	2654.5	3309.8	2590.3 ± 698.96	2681.9	1997.7	2681.9	3035.4	4533.9	2424.4 ± 659.57	2352.9	1997.7	2352.9	2847.5	4533.9	0.251
**Calcium (mg)**	642.4 ± 283.65	607.1	435.8	607.1	749.817	1328.2	742.9 ± 22.81	734.2	563.2	734.2	914.4	1256.4	695.6 ± 255.80	674.9	507.8	674.9	851.8	1328.2	0.109
**Magnesium(mg)**	244.1 ± 80.97	222.5	202.1	222.5	311.2	426.5	243.6 ± 58.64	236.6	208.1	236.6	274.5	376.1	243.9 ± 68.93	233.6	204.8	233.6	282.3	426.5	0.798
**Phosphorus (mg)**	1021.1 ± 274.79	968.5	805.3	968.5	1237.6	1623.9	1138.0 ± 211.49	1117.1	943.4	1117.1	1312.9	1612.2	1083.1 ± 246.71	1070.2	892.6	1070.2	1256.3	1623.9	0.135
**Iron (mg)**	9.7 ± 2.50	8.9	8.3	8.9	11.4	15.8	10.4 ± 3.08	9.7	8.8	9.7	11.6	17.9	10.1 ± 2.80	9.3	8.6	9.3	11.4	17.9	0.574
**Zinc (mg)**	8.4 ± 1.72	8.1	7.0	8.1	9.8	12.0	9.6± 2.07	9.3	8.2	9.3	10.5	13.5	9.1± 1.99	8.9	7.6	8.9	10.1	13.5	0.070

*p < 0.05

The daily energy, macro- and micronutrient intakes of children with diabetes according to HbA1c (%) level were compared with the Turkey Dietary Guideline 2022 [22], and no deficiency in energy, protein, carbohydrate, vitamin B1, vitamin B2, vitamin B3, vitamin B6, folate, sodium, potassium or iron consumption was observed. Vitamin A (5.6%), vitamin E (5.6%), vitamin C (5.6%), calcium (22.2%), and dietary fiber (5.6%) consumption was inadequate in children with an HbA1c level > 8, and vitamin C (6.3%), calcium (18.8%), dietary fiber (6.3%), and zinc (6.3%) consumption was inadequate in children with an HbA1c level < 8; morever, no statistically significant difference was found between the groups (Table 4).

**Table 4:** Average estimated percentages of daily energy, macro and micronutrient intakes of children with type 1 diabetes according to HbA1c (%) level according to TÜBER (2022)

Energy and nutrients		HbA1c <%8 (*n* = 16)		HbA1c >%8 (*n* = 18)		TOTAL (*n* = 34)		
		*N*	%	*N*	%	*N*	%	***P***
Energy (kcal)	<%50	-	-	-	-	-	-	0.086
	%50<%100	11	68.8	7	38,9	18	44.3	
	≥%100	5	31.2	11	61.1	16	45.7	
Protein (g)	<%50	-	-	-	-	-	-	0.164
	%50<%100	2	12.5	-	-	2	5.9	
	≥%100	14	87.5	18	100	32	94.1	
Carbohydrate (g)	<%50	-	-	-	-	-	-	0.810
	%50<%100	3	18.8	4	22.2	7	20.6	
	≥%100	13	81.2	14	77.8	27	79.4	
Dietary fibre (g)	<%50	1	6.2	1	5.6	2	5.8	0.420
	%50<%100	5	31.3	9	50.0	14	41.3	
	≥%100	10	62.5	8	44.4	18	52.9	
Vitamin A (µg)	<%50	-	-	1	5.6	1	2.9	0.615
	%50<%100	5	31.2	2	11.1	7	20.5	
	≥%100	11	68.8	15	83.3	26	76.6	
Vitamin E (mg)	<%50	-	-	1	5.6	1	2.9	0.804
	%50<%100	7	43.7	5	27.7	12	35.4	
	≥%100	9	56.3	12	66.7	21	61.7	
Thiamine (mg)	<%50	-	-	-	-	-	-	-
	%50<%100	-	-	-	-	-	-	
	≥%100	16	100	18	100	34	100	
Riboflavin (mg)	<%50	-	-	-	-	-	-	0.346
	%50<%100	7	43.8	5	27.8	12	35.2	
	≥%100	9	56.2	13	72.2	22	64.8	
Niacin (mg)	<%50	-	-	-	-	-	-	0.134
	%50<%100	4	25.0	1	5.6	5	14.7	
	≥%100	12	75.0	17	94.4	29	85.3	
Vitamin B6 (mg)	<%50	-	-	-	-	-	-	0.507
	%50<%100	7	43.7	10	55.5	17	50.0	
	≥%100	9	56.3	8	44.5	17	50.0	
Folate (µg)	<%50	-	-	-	-	-	-	0.559
	%50<%100	6	37.5	5	27.8	11	32.3	
	≥%100	10	62.5	13	72.2	23	67.7	
Vitamin 12 (µg)	<%50	1	6.3	-	-	1	2.9	0.386
	%50<%100	2	12.5	2	11.1	4	11.7	
	≥%100	13	81.2	16	88.9	29	85.4	
Vitamin C (mg)	<%50	1	6.3	1	5.6	2	5.8	0.948
	%50<%100	4	25.0	5	27.7	9	26.4	
	≥%100	11	68.7	12	66.7	23	67.8	
Calcium (mg)	<%50	3	18.8	4	22.2	7	20.5	0.598
	%50<%100	10	62.4	12	66.6	22	64.7	
	≥%100	3	18.8	2	11.2	5	14.8	
Iron (mg)	<%50	-	-	-	-	-	-	0.346
	%50<%100	9	56.2	13	72.2	22	64.7	
	≥%100	7	43.8	5	27.8	12	35.3	
Zinc (mg)	<%50	1	6.3	-	-	1	3.0	0.779
	%50<%100	6	37.5	10	55.6	16	47.0	
	≥%100	9	56.2	8	44.4	17	50.0	

Serum OC (ng/ml) (24.2 ± 16.92), levels were higher, and serum TOS (µmol/L) (8.7 ± 6.16) values were lower in individuals with HbA1C < 8% than those with HbA1C > 8%. Serum P1NP (ng/ml) (336.7 ± 294.30) levels were higher in individuals with HbA1C < 8% than those with HbA1C > 8% but not statistically significant (Table 5).

**Table 5:** Biochemical findings of children according to HbA1c (%) level

Variable	**HbA1c <%8 ( ** ***n*** ** = 16)**						**HbA1c >%8 (** ***n*** ** = 18)**						*P*
	$\bar x$ ±SD	Median	Q1	Q2	Q3	Q4	$\bar x$ ±SD	Median	Q1	Q2	Q3	Q4	
**TAS (mmol/L)**	1.6 ± 0.17	1.6	1.5	1.6	1.8	1.9	1.5 ± 0.12	1.5	1.5	1.5	1.7	1.8	0.064
**TOS (µmol/L)**	8.7 ± 6.16	7.8	4.2	7.8	9.6	23.8	9.5 ± 5.60	8.6	4.3	8.6	13.1	25.0	0.039*
**OC (ng/ml)**	24.2 ± 16.92	19.7	18.2	19.7	24.4	79.6	22.0 ± 6.21	20.4	18.7	20.4	25.0	36.6	0.002*
**P1NP (ng/ml)**	336.7 ± 294.30	263.4	172.8	263.4	339.5	1271.0	313.7 ± 186.46	224.3	188.9	224.3	402.5	826.0	0.117
**ALP (IU/L)**	284.1 ± 77.71	284.0	227.5	284.0	351.5	412.0	251.6 ± 75.01	254.5	186.7	254.5	327.5	359.0	0.191
**Vitamin D (IU)**	21.6 ± 8.34	21.7	19.3	21.7	28.7	32.6	22.5 ± 10.74	22.3	13.6	22.3	28.2	46.4	0.945

*p < 0.05, TAS: Total Antioxidant Status, TOS: Total Oxidative Stress, OC: Osteocalcin, P1NP: Procollagen Type-1 N-terminal Propeptide, ALP: Alkaline Phosphatase

The effect of the serum TOS level (µmol/L) on bone turnover biomarkers was evaluated by multiple regression analysis, and serum the TOS (µmol/L) and P1NP (ng/ml) levels were negatively correlated (*p* < 0.05) (Table 6).

**Table 6:** Multiple regression analysis for the effect of serum TOS (µmol/L) level on bone biomarkers

Variable	Beta	T	*P*	95% confidence interval	
**P1NP (ng/ml)**	-0.005	2.481	0.024*	0.001	0.010
**OC (ng/ml)**	0.014	0.382	0.707	-0.061	0.088
**ALP (IU/L)**	0.003	1.001	0.331	-0.003	0.009
**Vitamin D (IU)**	0.008	0.291	0.775	-0.048	0.063
**TAS (mmol/L)**	-1.501	2.388	0.029*	0.175	2.827

*p* < 0.05, P1NP: Procollagen Type-1-N-Terminal Propeptide, OC: Osteocalcin, ALP: Alkaline Phosphatase, TAS: Total Antioxidant Status, TOS: Total Oxidative Stress

## Discussion

In this study, serum TOS levels were greater and OC levels were lower in children with HbA1c levels > 8% than in those with HbA1c levels < 8%. A negative correlation was shown between serum TOS and P1NP levels.

Many studies in the literature have hypothesized that the cause of impaired bone turnover in diabetes patients is decreased bone turnover rather than increased bone resorption [23, 24]. Madsen et al. [5] evaluated OC, P1NP, and C-terminal cross-linked telopeptide of type-1 collagen (CTX) levels in individuals with T1DM aged 7.7–17.5 years and reported that these individuals had lower levels than did the reference population and that CTX levels were negatively associated with HbA1c levels. In another study, Madsen et al. [25] examined OC, P1NP, and CTX levels in 99 individuals with T1DM 3 months after diagnosis and three times at 6-month intervals and showed that bone destruction increased and bone turnover decreased in the first year after T1DM diagnosis. Vora et al. [26] evaluated dual-energy X-ray absorptiometry (DXA), peripheral quantitative computed tomography (pQCT), plantar fascia thickness, and microvascular complications in 64 adolescents with a duration of T1DM > 10 years and found site-specific low bone density in the upper and lower extremities. Eckert et al. [27] compared T1DM patients aged ≤ 25 years with a history of bone fracture with T1DM patients without a history of bone fracture and reported that bone fractures were more common at an earlier age in T1DM patients than in the general population and that the risk of fracture was associated with HbA1c levels. El Amrousy et al. [10] examined the serum ALP, P1NP, urinary deoxypyridinoline (DPD), glutathione, superoxide dismutase (SOD), and malondialdehyde (MDA) levels as bone biomarkers and glutathione, superoxide dismutase (SOD), and malondialdehyde (MDA) levels as OS markers in 40 T1DM patients and 40 control subjects aged < 18 years and found a relationship between oxidative stress and bone turnover biomarkers in patients with T1DM. Similarly, Heilman et al. [28] compared 30 children with T1DM (4.7–18.6 years) and healthy control subjects (4.7–18.6 years old) in terms of bone mineral density, glycemic control, OS (intercellular adhesion molecule-1 [ICAM-1]) and inflammation (high sensitivity C-reactive protein [hs-CRP], and urinary 8-iso prostaglandin F2a [F2-IsoPs]). They evaluated dietary calcium (Ca) intake and showed that poor glycemic control, increased OS, and inflammation were associated with low bone mineral density in children with T1DM.

Heilman et al. [28] evaluated the effect of Ca consumption on bone mineral density (BMD) in children with T1DM and healthy children. Low BMD was found in individuals with T1DM, and there was no difference between the groups in terms of Ca consumption. In their study, they evaluated only one nutrient, Ca. In addition to Ca, micronutrients such as magnesium, vitamin C, zinc, and iron have positive effects on bone turnover because they are cofactors for enzymes involved in bone metabolism and collagen synthesis [29]. In this study, the frequency of food consumption by children was evaluated, and macro- and micronutrient consumption was evaluated instead of the consumption of a single nutrient. The food consumption frequency method is an easy and accurate method for assessing daily food consumption. However, participant memory is required for accurate reporting, and certain nutrients may be overestimated or underestimated due to social factors and prejudice [30]. In this study, to eliminate these risks, the frequency of children’s food consumption was evaluated by an expert dietician. When the children were classified according to their HbA1c levels, protein consumption was found to be greater in the group with HbA1c levels > 8% than in the group with HbA1c levels < 8%; however, the percentage of protein in the diet did not differ between the groups, and the consumption of all the other nutrients was similar. In this study, the fact that nutrient consumption was generally similar provided a clearer picture of the effect of HbA1c and TOS levels on bone biomarkers independent of nutrient consumption.

In a study, it was stated that a meal pattern including more than one small meal should be supported in type 1 diabetes patients, but an increase in blood glucose variability can be expected with an increase in the number of meals eaten [31]. In our study, when dietary habits were evaluated, no difference was found according to the HbA1c level, and the majority of the individuals in both groups were found to comply with the meal pattern.

In all the studies in the literature, individuals with T1DM were compared with healthy controls, and the presence of T1DM was defined as a risk factor for low BMD. In this study, unlike in the literature, instead of including a healthy control group, children with T1DM were classified according to their HbA1c levels, and the effects of metabolic control and compliance with medical nutrition therapy on bone biomarkers were investigated. Serum OC levels were lower in children with HbA1c levels > 8% than in children with HbA1c levels < 8%, while no difference was found in ALP and P1NP levels, indicating that good metabolic control in T1DM patients may be protective for bone health.

Some studies [10, 32–34] showed that P1NP levels decreased as HbA1c levels increased. However in this study, there was no difference in the serum ALP levels between groups with different BMDs. This difference may be related to the evaluation of hepatic or intestinal ALP levels rather than bone-specific ALP levels [35].

In studies comparing serum vitamin D levels between individuals with T1DM and healthy control subjects, diabetic individuals were found to have lower vitamin D levels [36–38]. Bouichrat et al. [39] reported a negative correlation between HbA1c and vitamin D levels in individuals with T1DM. In this study, there was no difference in the serum vitamin D levels between patients with an HbA1c level > 8% and those with an HbA1c level < 8%.

Oxidative stress not only increases osteoclastogenesis but also inhibits osteoblast differentiation and thus bone turnover [40]. Studies evaluating the effect of OS on bone biomarkers in children with T1DM are limited in the literature, and SOD, MDA, and ICAM-1 were evaluated as OS indicators [10, 28]. However, it is not recommended to measure different oxidant and antioxidant molecules separately when assessing oxidative stress levels, as these methods may cause overlapping and imprecise results and have high costs; instead, TOS measurement may be more reliable, sensitive, and stable [15]. In this study, the TOS level was used as a reference indicator of oxidative stress. Serum TOS levels were found to be greater in patients with HbA1c levels > 8% than in those with HbA1c levels < 8%. In this study, a negative correlation between serum TOS and TAS levels was also detected, and low TAS levels in children with an HbA1c level > 8% was considered a risk factor contributing to high TOS levels. In addition, a negative correlation was found between TOS and serum P1NP levels in this study. Like our findings, Amrousy et al. [10] also reported a negative correlation between P1NP and serum malondialdehyde levels, which they evaluated as an indicator of OS in children with T1DM. P1NP has very low circadian and biological variation, is not affected by food consumption, and is reported to be stable in serum after venipuncture [41].

In previous studies [42, 43], the effect of metabolic control on bone biomarkers was evaluated, but serum TAS levels and food consumption, which are indicators of defense mechanisms, were not evaluated. Our results showed that serum TOS levels were greater and OC levels were lower, and TOS levels had an effect on P1NP in T1DM patients without metabolic control.

In conclusion, impaired metabolic control as a result of nonadherence to medical nutrition therapy leads to high TOS levels and negatively affects bone biomarkers.

The limitations of this study include the small sample size, the use of HbA1c levels alone as an indicator of metabolic control, and the wide age range. Studies in which compliance with dietary treatment is evaluated in detail and the age group is planned to be within a narrower range are needed.

## Data Availability

The data that support the findings of this study are available from the corresponding author upon reasonable request.

## Abbreviations

AGE: Glycation end products
ALP: Alkaline phosphatase
BMI: Body Mass Index
BMD: Bone mineral density
Ca: Calcium
CTX: C-terminal cross-linked telopeptide of type-1 collagen
DPD: Deoxypyridinoline
DXA: Dual energy X-ray absorptiometry
F2-IsoPs: 8- iso prostaglandin F2a
HbA1C: Glycated hemoglobin
Hs-CRP: High sensitivity C-reactive protein
ICAM-1: Intercellular adhesion molecule-1
IGF-1: Insulin-like growth factor-1
MDA: Malondialdehyde
OC: Osteocalcin
OS: Oxidative stress
PQCT: Peripheral quantitative computed tomography
P1NP: Procollagen Type-1-N-Terminal Propeptide
ROS: Reactive oxygen species
SOD: Superoxide dismutase
TAS: Total antioxidant status
TOS: Total oxidative status
T1DM: Type 1 diabetes mellitus
